# Automatic ICD-10 Coding and Training System: Deep Neural Network Based on Supervised Learning

**DOI:** 10.2196/23230

**Published:** 2021-08-31

**Authors:** Pei-Fu Chen, Ssu-Ming Wang, Wei-Chih Liao, Lu-Cheng Kuo, Kuan-Chih Chen, Yu-Cheng Lin, Chi-Yu Yang, Chi-Hao Chiu, Shu-Chih Chang, Feipei Lai

**Affiliations:** 1 Graduate Institute of Biomedical Electronics and Bioinformatics National Taiwan University Taipei Taiwan; 2 Department of Anesthesiology Far Eastern Memorial Hospital New Taipei City Taiwan; 3 Department of Internal Medicine National Taiwan University Hospital National Taiwan University College of Medicine Taipei Taiwan; 4 Department of Internal Medicine Far Eastern Memorial Hospital New Taipei City Taiwan; 5 Department of Medical Affairs Far Eastern Memorial Hospital New Taipei City Taiwan; 6 Department of Healthcare Administration Oriental Institute of Technology New Taipei City Taiwan; 7 Department of Information Technology Far Eastern Memorial Hospital New Taipei City Taiwan; 8 Section of Cardiovascular Medicine Cardiovascular Center Far Eastern Memorial Hospital New Taipei City Taiwan; 9 Section of Health Insurance Department of Medical Affairs Far Eastern Memorial Hospital New Taipei City Taiwan; 10 Medical Records Department Far Eastern Memorial Hospital New Taipei City Taiwan; 11 Department of Computer Science and Information Engineering National Taiwan University Taipei Taiwan; 12 Department of Electrical Engineering National Taiwan University Taipei Taiwan

**Keywords:** natural language processing, deep learning, International Classification of Diseases, Recurrent Neural Network, text classification

## Abstract

**Background:**

The International Classification of Diseases (ICD) code is widely used as the reference in medical system and billing purposes. However, classifying diseases into ICD codes still mainly relies on humans reading a large amount of written material as the basis for coding. Coding is both laborious and time-consuming. Since the conversion of ICD-9 to ICD-10, the coding task became much more complicated, and deep learning– and natural language processing–related approaches have been studied to assist disease coders.

**Objective:**

This paper aims at constructing a deep learning model for ICD-10 coding, where the model is meant to automatically determine the corresponding diagnosis and procedure codes based solely on free-text medical notes to improve accuracy and reduce human effort.

**Methods:**

We used diagnosis records of the National Taiwan University Hospital as resources and apply natural language processing techniques, including global vectors, word to vectors, embeddings from language models, bidirectional encoder representations from transformers, and single head attention recurrent neural network, on the deep neural network architecture to implement ICD-10 auto-coding. Besides, we introduced the attention mechanism into the classification model to extract the keywords from diagnoses and visualize the coding reference for training freshmen in ICD-10. Sixty discharge notes were randomly selected to examine the change in the F_1_-score and the coding time by coders before and after using our model.

**Results:**

In experiments on the medical data set of National Taiwan University Hospital, our prediction results revealed F_1_-scores of 0.715 and 0.618 for the ICD-10 Clinical Modification code and Procedure Coding System code, respectively, with a *bidirectional encoder representations from transformers* embedding approach in the Gated Recurrent Unit classification model. The well-trained models were applied on the ICD-10 web service for coding and training to ICD-10 users. With this service, coders can code with the F_1_-score significantly increased from a median of 0.832 to 0.922 (*P*<.05), but not in a reduced interval.

**Conclusions:**

The proposed model significantly improved the F_1_-score but did not decrease the time consumed in coding by disease coders.

## Introduction

The International Classification of Diseases (ICD) is a medical classification list released by the World Health Organization, which defines the universe of diseases, disorders, injuries, and other related health conditions and the classifying standard of diagnosis [1]. Since the first publication in 1893, the ICD has become one of the most important indexes in medical management systems, health insurance, or literature research.

At present, in most medical institutions, ICD-10 codes that are used in diagnostic related group subsidy for inpatients mainly rely on manual coding from a group of licensed and professional disease coders on a case-by-case basis, who spend a lot of time reading a multitude of medical materials. On the other hand, some other cases—especially outpatients—are coded by physicians.

Since the conversion from ICD-9 to ICD-10 in 2014, Taiwan has used the ICD-10 as the reference for diagnostic-related group subsidy. However, because of the complexity of the ICD-10 structure and coding rules such as the code orders, the inclusion and exclusion criteria, and the enormously increasing number of ICD-10 codes, ICD-10 coding work became much more laborious and time-consuming, even if a disease coder with professional abilities takes approximately 30 minutes per case on average. According to the analysis from *Handbook of Research on Informatics in Healthcare and Biomedicine*, the cost for adopting the ICD-10 system, including training of disease coders, physicians, and code users; initial and long-term loss of productivity among providers; and sequential conversion, is estimated to range from a 1-time cost of US $425 million to US $1.15 billion in addition to US $5-40 million per year in lost productivity [2].

Previous studies had built a model for the ICD-9 system. In 2008, Farkas and Szarvas [3] utilized a rule-based approach querying other reference tools to implement the ICD auto-coding task. However, compared to ICD-9, ICD-10 contains more than 60,000 codes. Building a rule-based automatic system is labor-intensive and time-consuming. In addition, the entirety of the rules of the ICD-10 system is complicated even for disease coders. For the aforementioned reasons, recent studies have emphasized on deep learning– and natural language processing (NLP)–related approaches; for instance, Zhang et al [4] used a gated recurrent unit (GRU) network with content-based attention to predict medication prescriptions on the basis of the disease codes, and Wang et al [5] applied and compared NLP techniques such as Global Vectors (GloVe) in an electronic health record (EHR) data classification task.

In previous studies, we have already applied word to vectors (Word2Vec), an NLP method, in an ICD-10 auto-coding task and achieved an F_1_-score of 0.67/0.58 in Clinical Modification (CM)/Procedure Coding System (PCS). Furthermore, we also built an ICD-10 code recommendation system for ICD-10 users [6,7]. In this study, we made a comparison on most of the recent NLP approaches such as Word2Vec, embeddings from language models (ELMo), and bidirectional encoder representations from transformers (BERT). Furthermore, we introduced the attention mechanism to our classification model to visualize the word importance for training new coders in ICD-10 coding.

**Figure 1:**
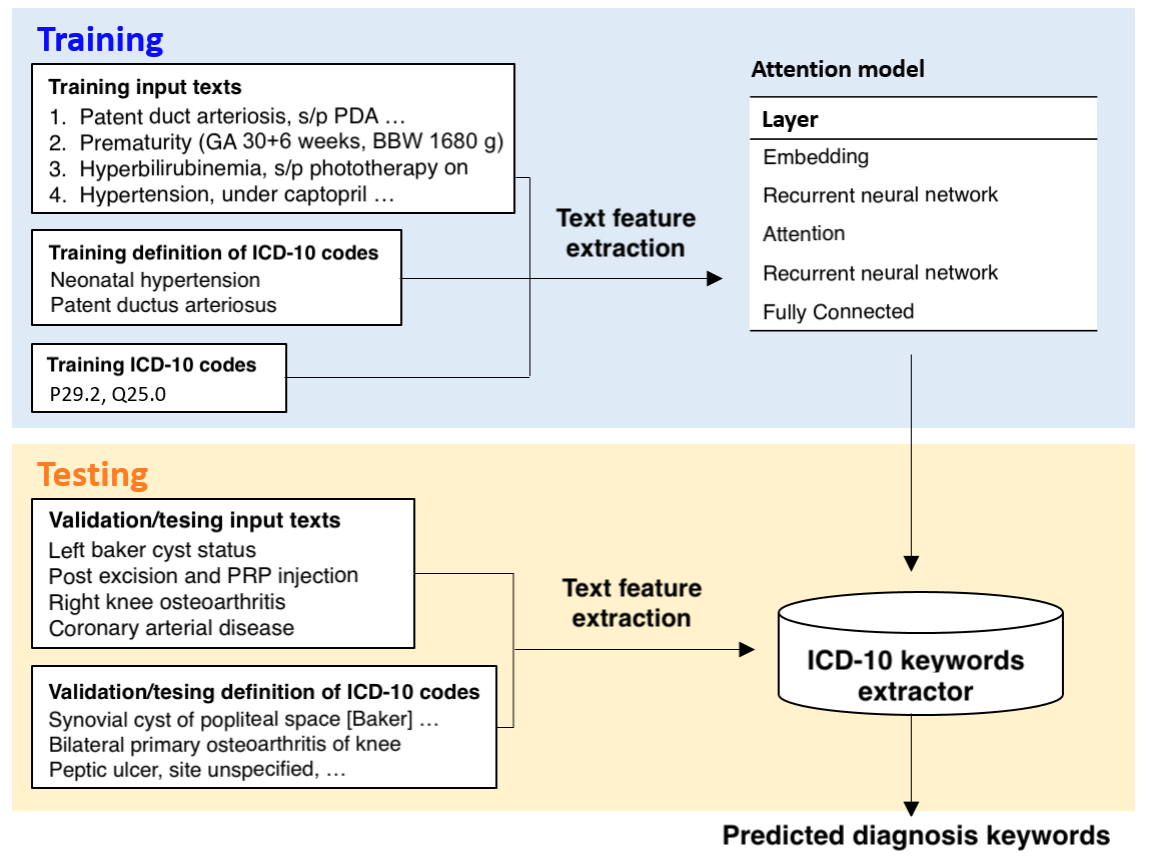
Training and validation process for the ICD-10 classification and attention models. BBW: birth body weight; GA: gestational age; PRP: platelet-rich plasma.

In the ICD classification framework illustrated in Figure 1, the left panel denotes the large amounts of free-text data written by physicians, which would be read and learned by the classifier in the right panel of the graph with supervised learning. Well-trained classifiers would be applied to predict the ICD-10 codes accurately for each patient. Furthermore, to distinguish the primary, secondary, or additional diagnosis, a sequential correction was conducted by coding the ICD-10 codes in a sequential format, using a sequence-to-sequence model followed by combining the classification coding results with the sequential order outcome.

The attention framework for paragraph highlighting is also illustrated in Figure 1. Different from the classification framework, the input data in the left panel include both the diagnoses and the corresponding ICD-10 definitions from the National Health Insurance Administration rather than using merely the diagnoses, and the output data in the right panel is the attention weight matrix extracted from the predicting process rather than the classification result. With a combination of these 2 methods, we constructed an ICD-10 auto-coding and training system to assist ICD-10 code users.

Our study aims at building an automatic ICD-10 coding and training system based on NLP technology, attention mechanism, and Deep Neural Network (DNN) models, which are applied for extracting information from EHR data, highlighting the key points from the extracted features, and implementing an ICD-10 classification task with sequential correction, respectively, for assisting all ICD-10 users.

## Methods

### Data Description

Our data were acquired from patients at National Taiwan University Hospital (NTUH) from January 2016 to July 2018. The ground-truth ICD-10 codes were annotated by the coders at NTUH. Data attributes and types include account IDs, type contents, course and treatment, and discharge diagnoses. The distribution of ICD-10 codes is shown in our previous study [7].

### System Architecture

**Figure 2:**
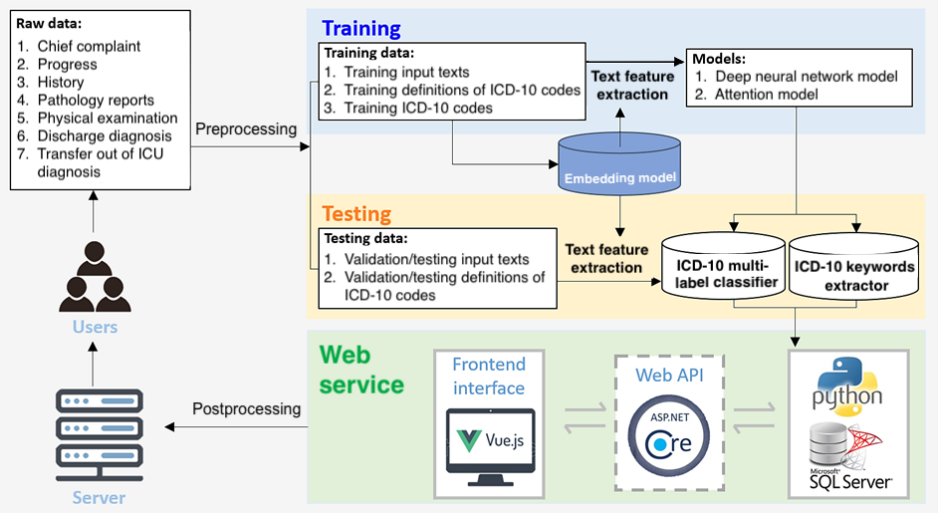
Complete framework of the ICD-10 auto-coding and training system. API: application programming interface; ICU: intensive care unit.

The entire process of the system constructing framework is composed of data processing, feature extracting, model constructing, model training, and web service building. To detail and visualize the ICD-10 web service clearly in this study, the complete workflow of the ICD-10 coding and training system is illustrated in Figure 2.

### Data Processing

#### Preprocessing

Preprocessing, including the removal of Chinese words, null or duplicate elements, punctuation, stop words, and infrequent words, was applied before tokenization of the texts. The basic preprocessing methods were applied using the Natural Language Toolkit [8] and Scikit-Learn [9] library. We then randomly split the data set at a 9:1 ratio into training and validation sets with the Scikit-Learn library.

#### Postprocessing

In ICD-10 coding, combination codes remain an intractable issue because, in some cases, disease coders cannot—and should not—assign multiple diagnosis codes when a single combination code clearly identifies all aspects of the patient’s diagnosis [10]. In this study, a user-defining panel is provided in the auto-coding system to deal with combination codes by replacing the incorrect outcomes, where the combination codes were either predicted incorrectly or separated into 2 different codes on the basis of the given codes.

### Feature Extraction

During feature extraction, we applied NLP techniques, including GloVe [11], Word2Vec [12], ELMo [13], BERT [14], and single head attention recurrent neural network (SHA-RNN), to convert the word contexts to numerical data and extract word and contextual information. Except for the BERT-based pretrained weight, we also attempted clinicalBERT [15] and BioBERT [16], which were trained with clinical notes from MIMIC-III, PubMed, and PubMed Central. Hyperparameters of the embedding models are attached in Table 1.

**Table 1:** Hyperparameters of word-embedding models.

Hyperparameters		Size/number
**Global Vector**		
	Word embedding size	100
**Word to Vectors**		
	Word embedding size	300
**Embeddings from Language Models**		
	Convolutional neural network char embedding size	50
	Convolutional neural network word embedding size	100
	Highway number	2
	Intermediate size	512
**Bidirectional encoder representations from transformers^a^**		
	Word embedding size	768
	Sentence embedding size	768
	Position embedding size	768
	Intermediate size	3072
	Attention head number	12
	Hidden layer number	12
	Dropout	0.1
**Single head attention recurrent neural network**		
	Word embedding size	1024
	Hidden size	1024
	Layer number	4

^a^Clinical bidirectional encoder representations from transformers (BERT) and BERT for biomedical text mining shared the same hyperparameters with BERT.

### Classification Model

**Figure 3:**
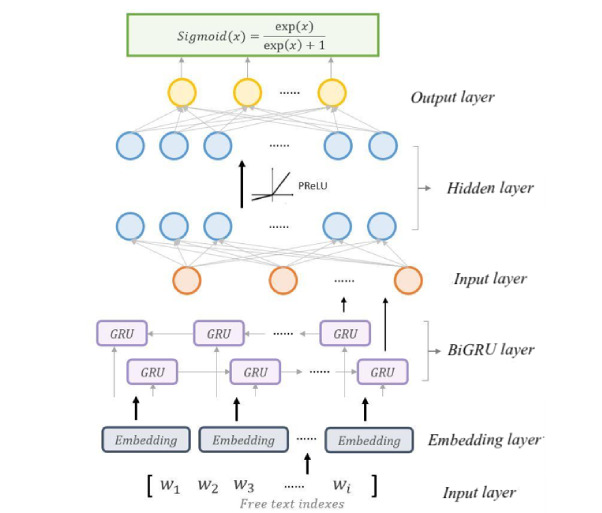
Architecture of the Deep Neural Network classification model. BiGRU: Bidirectional Gated Recurrent Unit; GRU: Gated Recurrent Unit; PReLU: Parametric Rectified Linear Unit.

The classification model was constructed with 4 neural network layers, including RNN and fully connected neural network (FCNN), where the hyperparameters are shown in Table 2 and the architecture is shown in Figure 3. The first layer is the word embedding layer, which transforms the tokenized word list input into word vectors. The second layer is a bidirectional GRU (BiGRU) layer [17]. The remaining 2 layers are fully connected layers, where the final fully connected layer should be set to the size of the dimension we expect to predict. In our case, we conducted 2 classification tasks, including whole label classification for CM and PCS with 14,602/9780 labels of CM/PCS in NTUH data records in total. Hence, the final fully connected layer size should be set to 14,602 and 9780 dimensions, respectively. To make a comparison, a classification model with only 1 fully connected layer— fully connected layer 2—was used as the baseline model. In addition, the attention mechanism based on the Bahdanau [18] attention model was introduced to our classification model to further extract the keywords for ICD-10 coding by computing the weight information of context—ICD title–vector pairs; that is, the importance of the information with respect to the current target word.

**Table 2:** Hyperparameters of the classification models.

Hyperparameters	Size
Bidirectional GRU^a^ layer	256
Fully connected layer 1	700
Fully connected layer 2 CM/PCS^b^	14,602/9780
Dropout	0.2

^a^GRU: Gated Recurrent Unit.

^b^CM/PCS: Clinical Modification/Procedure Coding System.

### Model Assessment

Micro F_1_-score is the harmonic mean of recall and precision, which are the sum of the number of true-positive results divided by sum of the number of all positive results and the sum of the number of true-positive results divided by the sum of the number of all relevant samples, respectively. The micro F_1_-score considers the number for each label while calculating the recall and precision; hence, it is appropriate for evaluating the performance of a multi-label classification task with imbalanced data set.

For realistic application in the auto-coding system, recall@K, which calculates the proportion of correct answers in the first K prediction results returned by the classifier, was also applied for validating the model’s performance. In our case, considering the limitation of the quantity of CM and PCS codes, 20 was chosen as the K value.

### ICD-10 Coding and Training System Framework

**Figure 4:**
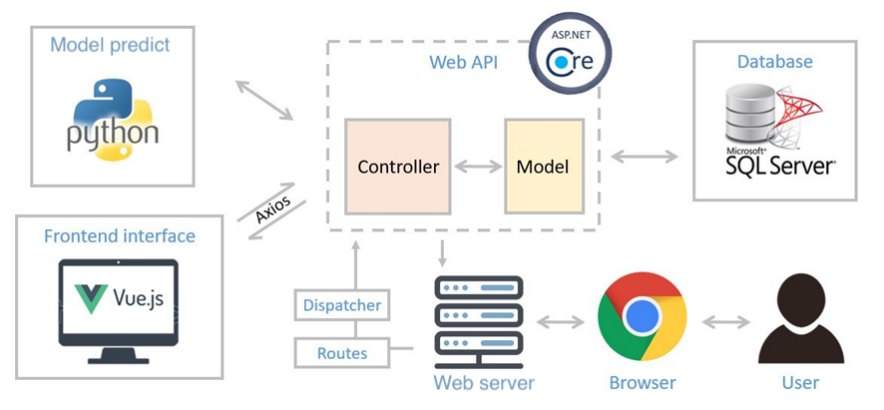
System architecture of the ICD-10 auto coding and training web service. API: application programming interface.

An ICD-10 auto-coding and training system prototype was constructed with python3, ASP.NET Core 2.2 MVC, SQL Server, and Vue.js. Whenever a user performs an action, such as typing a discharge diagnosis or retrieving information from a database on the frontend interface built with Vue.js, the axios, a promise-based HTTP client for the browser and node.js, would call for the Web application programming interface in the backend built with ASP.NET Core 2.2 MVC to send the case information to the backend for predicting and processing via python3 or to the database for data preservation in SQL Server. The complete system framework is illustrated in Figure 4. In ICD-10 Coder and Trainer, with the discharge diagnosis as the data input, the top 20 related ICD-10-CM/PCS codes and the importance of each word related to the corresponding code would be returned to all ICD-10 users for auxiliary.

### Comparing the Time Consumed and the F_1_-Score With and Without the Auto-Coding System

We collected 60 discharge notes from February 2021 from the Far Eastern Memorial Hospital (New Taipei City, Taiwan) randomly. Nine coders participated in this experiment. The most experienced coder provided the ground truth. The other 8 coders were divided into 4 groups, and each case assigned to each group could be coded by 2 coders. There are 2 parts in this experiment. In part 1, we only provided medical record numbers, and the coders coded the randomly assigned medical records on a daily basis. Each group was assigned a different set of 10 cases. In part 2, we provided medical record numbers and ICD codes predicted by our best DNN classification model. Each group was randomly assigned 5 cases. We compared the time consumed and the F_1_-score between parts 1 and 2 and performed a paired samples Wilcoxon signed-rank test. A 2-tailed *P*<.05 was considered significant. Furthermore, a questionnaire was designed to collect coders’ opinions on this system.

## Results

### ICD-10-CM Whole Label Classification

In the NTUH data set, the complete ICD-10-CM codes (ie, CM codes with 3-7 characters) corresponding to the discharge diagnosis records comprise 14,602 labels in total. The best DNN classification model based on BERT embedding and FCNN with BiGRU could achieve an F_1_-score of 0.715 and recall@20 of 0.873. Table 3 shows all comparisons of the whole label classification. Classification results with different BERT pretrained models show no significant effect on performance in both of baseline and BiGRU models.

**Table 3:** F_1_-score and Recall@20 of all embedding models in the International Classification of Diseases-10 Clinical Modification.

Embedding model	Baseline F_1_-score	F_1_-score	Recall@20
Word to Vectors	0.355	0.680	0.873
Global Vectors	0.220	0.635	0.836
Embeddings from Language Models	0.633	0.631	0.852
Bidirectional encoder representations from transformers–based	0.715	0.710	0.869
Clinical bidirectional encoder representations from transformers model	0.712	0.714	0.869
Bidirectional encoder representations from transformers for biomedical text mining	0.709	0.701	0.863
Single Head Attention Recurrent Neural Network	0.402	0.570	0.835

### ICD-10-PCS Whole Label Classification

In the ICD-10-PCS whole label classification task, the complete ICD-10-PCS code (ie, PCS codes with 7 characters) corresponding to discharge diagnosis records comprised 9513 labels. Progress and discharge diagnosis were applied for training the DNN model. The results summarized in Table 4 imply that our best DNN classification model based on BERT embedding and FCNN with BiGRU could achieve an F_1_-score of 0.618 and a recall@20 of 0.887.

**Table 4:** F_1_-score and recall@20 of all embedding models in the International Classification of Diseases-10 Procedure Coding System.

Embedding model	Baseline F_1_-score	F_1_-score	Recall@20
Word to Vectors	0.278	0.580	0.850
Global Vectors	0.120	0.520	0.841
Embeddings from Language Models	0.547	0.557	0.874
Bidirectional encoder representations from transformers–based	0.618	0.611	0.880
Clinical bidirectional encoder representations from transformers model	0.596	0.615	0.887
Bidirectional encoder representations from transformers for biomedical text mining	0.611	0.613	0.880
Single Head Attention Recurrent Neural Network	0.269	0.527	0.879

### ICD-10 Classification With Attention

**Figure 5:**
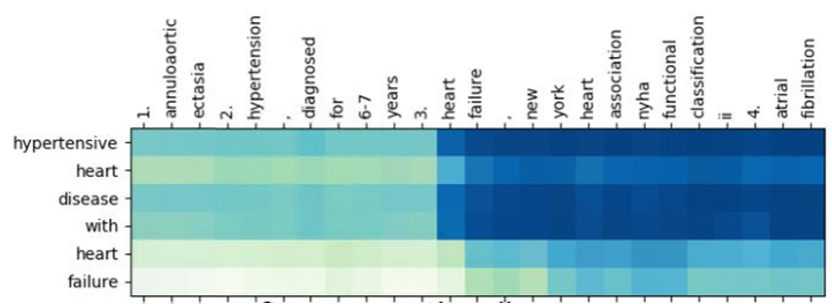
Visualization of attention weights.

By introducing the attention mechanism into the classification model, the relation and importance between word pairs could be computed and visualized. For instance, for 2 sentences, “He had coronary artery disease. Also, he got fever.” and “A heart disease,” weight information for the word “heart” might focus on “coronary” or “artery.” Hence, by extracting the attention weights of the diagnoses and ICD-10 definitions, how coders focus on the words within diagnoses during the ICD-10 coding process could be well understood (Figure 5). Furthermore, the extracted diagnosis attention weights and the corresponding ICD-10 code could be visualized by highlighting the key words, the weight of which would be higher than a certain threshold, for training a new coder in disease coding. By considering all positive cases and negative sampling up to 40 cases in total, the classification model with the attention mechanism could achieve an F_1_-score of 0.86.

### ICD-10 Coding and Training System Framework

The objective of this study is to build an ICD-10 auto-coding and training system for assisting disease coders to elevate their work efficiency and coding accuracy. An ICD-10 auto-predicting interface with discharge diagnosis as the reference is available on the internet [19] for accelerating the coding efficiency. The DNN model executed by the python script would return the top 20 ICD-10-CM and ICD-10-PCS codes with a recall@20 of 0.87 and 0.88, respectively. The predicting process of each case takes less than 30 seconds, which drastically shortens the coding time of 30 minutes per case on average. In addition, training for ICD-10 coding is also provided under the training tab. Given a paragraph of discharge diagnosis, the key words to support the code could be highlighted by clicking on the target code.

**Figure 6:**
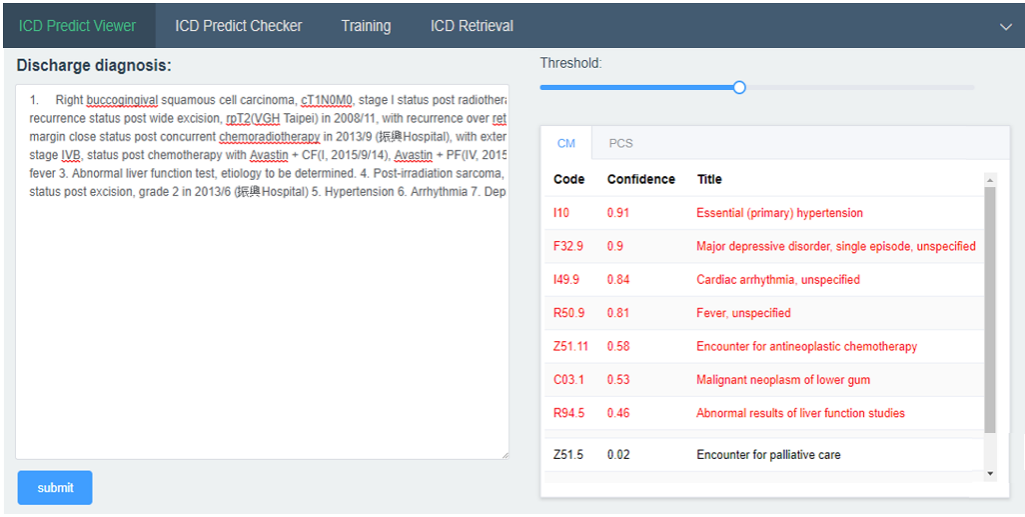
ICD-10 auto-coding panel.

**Figure 7:**
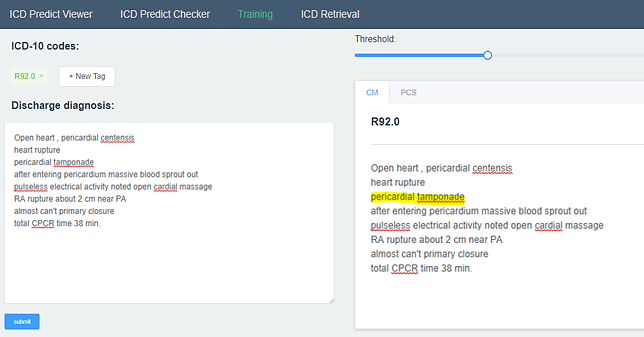
ICD-10 auto-training panel.

**Figure 8:**
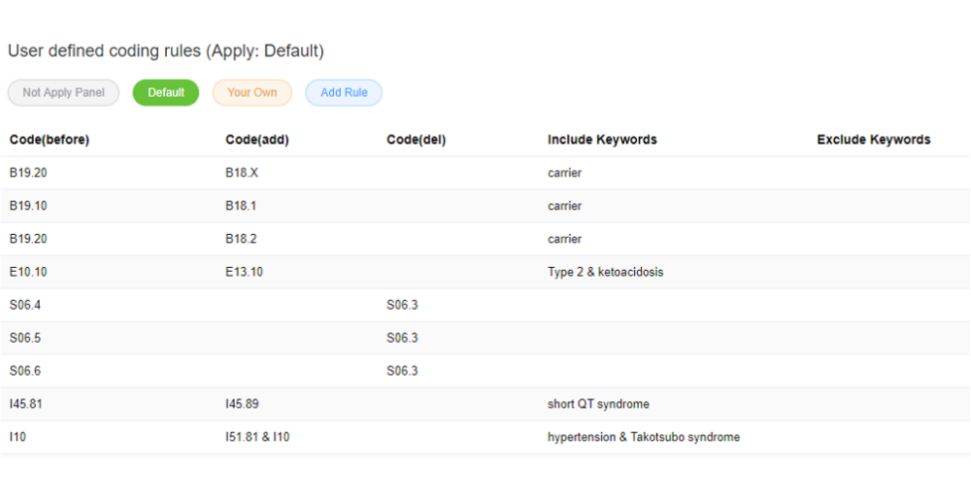
Postprocessing user defining panel.

To make the prediction more flexible and adaptable to disease coders in different hospitals, postprocessing rules for dealing with exceptions, such as combination codes and hospital consensus, could be defined under the rule definition panel. Users could apply the default setting or build their own setting to apply the specific coding style. The ICD-10 auto-coding, training, and rule defining panels are shown in Figures 6, 7, and 8 respectively.

### Time Consumed and F_1_-Score With and Without the Auto-Coding System

The ICD-10 auto-coding system with our best DNN classification model significantly improved the coders’ mean F_1_-score from a median of 0.832 to 0.922 (*P*<.05) but did not decrease their mean coding time (*P*=.64), as shown in Table 5. The questionnaire revealed that a coder took approximately 20-40 minutes on average to code a case, and 62.5% of coders are willing to use this system in their work. This system might potentially help them not only increase the accuracy of ICD-coding but also save their time.

**Table 5:** Time consumed and the F_1_-score with and without the auto-coding system.

Coder	Mean time consumed in part 1^a,b^ (minutes:seconds)	Mean time consumed in part 2^c,d,e^ (minutes:seconds)	Mean F_1_-score in part 1^a,f^	Mean F_1_-score in part 2^c,g,h^
1	07:49	05:11	0.801	0.893
2	08:19	06:01	0.900	0.960
3	04:57	06:16	0.980	0.951
4	05:02	07:32	0.867	0.950
5	06:23	05:18	0.766	0.978
6	05:23	03:53	0.652	0.892
7	05:45	05:25	0.815	0.838
8	05:33	06:43	0.848	0.827

^a^Without the auto-coding system.

^b^Median time consumed in part 1=5 minutes 39 seconds (95% CI 5 minutes 1 second to 7 minutes 54 seconds).

^c^With the auto-coding system.

^d^Median time consumed in part 2=5 minutes 43 seconds (95% CI 4 minutes 56 seconds to 6 minutes 52 seconds).

^e^Nonsignificant difference in the mean time consumed by coders between parts 1 and 2 of the study (2-tailed *P*=.64 derived from a paired samples Wilcoxon signed-rank test).

^f^Median F_1_-score in part 1=0.832 (95% CI 0.744-0.915).

^g^Median F_1_-score in part 2=0.922 (95% CI 0.836-0.963).

^h^Significant difference in mean F_1_-scores between parts 1 and 2 (2-tailed *P*<.05 derived from a paired samples Wilcoxon rank sum test).

## Discussion

### Principal Findings

Compared to a previous study on ICD-9 classification with 85,522 training data and an F_1_-score of 0.41 [20], our best DNN classification model based on the BERT embedding method and FCNN with BiGRU achieved an F_1_-score of 0.715 and recall@20 of 0.873. Comparing to the baseline model with only 1 fully connected layer, models with BiGRU showed better performance within the embedding approaches using fixed word embedding vectors. However, within embedding methods that are more flexible, such as BERT, the BiGRU classification model shows no significant effect on performance. This indicates that higher-level embedding techniques such as ELMo and BERT could certainly be able to sequentially consider the contextual semantics information; since they widely introduce the BiGRU and BiLSTM layers or other contextual information extraction methods within their model architectures. On the other hand, among all the embedding methods, BERT showed the best performance; however, it seems that initializing with different BERT pretrained weights has no significant influence on the classification results. However, the simplified BERT model SHA-RNN could only achieve 0.57 on the classification task and could not achieve over 0.41 on the baseline model. This might result from the lack of the corpus on training of the embedding model, comparing to BERT models which were trained with millions of articles from Bookcorpus, Wikipedia, etc; we only used our own discharge diagnosis records on SHA-RNN training. This implies the ability of the BERT model to learn and extract the information well in a specific field via only the fine-tuning process; thus, there is no need to train our BERT model from scratch with our own data set, but rather only to initialize with the pretrained weight and fine-tune with our own data set.

Another previous study compared BERT with other DNNs in ICD-10 auto-coding in nontechnical summaries of animal experiments. They achieved a micro F_1_-score of 73.02% with BioBERT, which is comparable to our results [21]. However, nontechnical summaries of animal experiments are not as complicated as the medical records we worked on and BioBERT could perform better than BERT in their data set, but no significant difference was observed in the medical records, as shown herein. Another study found that contextualized deep learning representation models including BERT and ELMo outperform noncontextualized representation models in discovering medical synonyms [22], which is consistent with our findings.

Our system improved the coder’s mean F_1_-score (*P*<.05) but did not decrease the mean coding time (*P*=.64). One of the explanations is that coders had not become familiar with this system yet, and the other explanation is that relatively simple cases were included in this experiment, which led them to take less than 20-40 minutes per case during their daily work, as they indicated in their questionnaire responses. The long-term effect of the ICD-10 auto-coding system should be investigated in the future to determine whether the coding time can be saved.

### Limitations

Our study has some limitations. First, our training data are derived from only 1 medical center. The performance in other hospitals could be affected by different writing habits, and different disease prevalence. Second, combination codes remain an intractable issue because in some cases, disease coders cannot and should not assign multiple diagnosis codes in cases where a single combination code clearly identifies all aspects of the patient’s diagnosis. In our results, the combination codes were either predicted incorrectly or separated into 2 different codes. In addition, there are multiple diagnoses that corresponded to multiple codes in order; that is, primary diagnosis, secondary diagnosis, tertiary diagnosis, etc [10]. However, the classification model could only give the probability of each code rather than the corresponding order. To resolve the problem while maintaining high performance in the classification task, we proposed a novel approach by combining the Seq2Seq model, which gives the code order. Finally, our system is still new to coders, and few coders have used it. After more users’ responses are collected, further analysis and modification can be performed to improve our system.

### Conclusions

In this study, an ICD-10 classification model developed using NLP and a deep learning model without any background knowledge from EHR data yielded an F_1_-score of 0.715 and 0.618 for CM and PCS, respectively. In addition, we built and released the platform for automated ICD-10 prediction and training based on our well-trained models for free to ICD-10 users worldwide and further shortened the coding time from 20-40 minutes to 30 seconds per case. Our platform can be found on the internet [19]. Our system can significantly improve coders’ F_1_-score in ICD-10 coding.

In future studies, we shall attempt to develop and provide other functions such as user feedback and auto-training with new input data to our model. ICD-10 codes in different hospitals with different coding styles will also be constructed in accordance with the amount of user information and prediction history records to improve the automated ICD-10 coding and training system further.

## Abbreviations

BERT: bidirectional encoder representations from transformers
BiGRU: Bidirectional Gated Recurrent Unit
BioBERT: bidirectional encoder representations from transformers for biomedical text mining
CM: Clinical Modification
DNN: Deep Neural Network
EHR: Electronic Health Records
ELMo: Embeddings from Language Models
FCNN: fully-connected neural network
GloVe: Global Vectors
GRU: Gated Recurrent Unit
ICD: International Classification of Diseases
NTUH: National Taiwan University Hospital
NLP: natural language processing
PCS: Procedure Coding System
SHA-RNN: Single Head Attention Recurrent Neural Network
Word2Vec: Word to Vectors
